# Effects of Different Pretreatments on the Nutrition, Flavor and Sensory Evaluation of Lactobacilli-Fermented Peach Beverages

**DOI:** 10.3390/foods14244303

**Published:** 2025-12-14

**Authors:** Qiaoyu Han, Jiechao Liu, Hui Liu, Qiang Zhang, Zhenzhen Lv, Dalei Chen, Wenbo Yang, Zhonggao Jiao

**Affiliations:** 1 Zhengzhou Fruit Research Institute, Chinese Academy of Agricultural Science, Zhengzhou 450009, Chinaliuhui@caas.cn (H.L.);; 2 Zhongyuan Research Center, Chinese Academy of Agricultural Science, Xinxiang 453000, China

**Keywords:** peach, *Lactobacillus* fermentation, polyphenols, antioxidant activity, carotenoids, volatile organic compounds

## Abstract

This study aimed to investigate the behavior and fermentation performance of *Lactobacillus* in peach purees and juice prepared using different pretreatments, and characterized the nutrition and flavor profiles of various fermented peach samples by using HPLC, HS-SPME-GC/MS and sensory evaluation. The findings showed that fermented peach products made from different raw material methods displayed distinct nutritional and sensory properties. The fermented CWP (crushing into puree with peel) had the highest total phenol content (145.20 μg/mL) and antioxidant activity (96.19 μg VC/mL), and fermented COP (crushing into puree without peel) was rich in carotenoids (1575.33 μg/100 mL), with β-carotene (1134.89 μg/100 mL) being the characteristic bioactive compound in this group. Moreover, fermented CWP and COP purees were also notable for their elevated aroma compounds, with total volatile organic compounds increasing 2.35 and 3.29 times after fermentation, respectively. However, fermented SWP juice (squeezing juice with peel) was primarily distinguished by polyphenol biotransformation, and had a similar polyphenol composition to the CWP group. These findings evidenced the advantages and characteristics of different peach matrices as raw materials for plant-based fermentation beverages, and offer strategies for developing functional probiotic fermented drinks.

## 1. Introduction

Fermentation is a traditional technique widely used in food processing that prolongs the shelf life of foods while enhancing their nutrition, flavors and functional properties [1]. Consuming fermented foods is recognized as an important way to obtain probiotics and metabolites, which offer health benefits to the host such as regulating gut microbiota, boosting free radical scavenging ability, strengthening the immune system and reducing blood cholesterol levels [2,3,4]. Recently, plant-based fermented beverages have attracted scientific interest due to shifts in dietary preferences, lower energy consumption and less greenhouse gas emissions [5,6]. According to statistics, the global market for fermented plant-based alternatives is expected to reach $422.26 million in 2026 [7].

Peach [*Prunus persica* (L.) Batsch] is grown worldwide and is highly favored by consumers because of its rich nutritional content and pleasant fragrance [8]. It has been investigated as a novel raw material for plant-based fermented beverages, with studies confirming its excellent fermentation performance as a growth medium for *Lactobacillus* spp. [9,10,11]. In our previous studies, *Lactiplantibacillus plantarum* 21802 was selected as the most suitable strain for grape juice fermentation, and confirmed that its fermentation product could enhance the level of free radical scavenging in mice [12]. In addition, the fermentation characteristics of Lactobacillus spp. were compared in strawberry puree and peach pulp, and further demonstrated that *Lactiplantibacillus plantarum* 21802 possessed potential application in fruit fermentation for enhancing the nutrition, flavor, and sensory and functional properties [11,13]. However, the specific composition of the matrix plays a critical role in designing fermented food and maximizing its functional benefits [14].

The pretreatment of raw material is an essential step during peach processing, such as peeling, crushing or squeezing, which determines the final product form and results in significant variations in the phytochemical composition of the products [15]. The composition and the content of phytochemicals were varied across different parts of the peach, and over 20% of phytochemicals in peaches are found in the peel. Compared with peach pulp, the peel presents higher levels of total phenols, total flavonoids and antioxidant capacity [8,16].

Considering peach peel’s edible and nutritive properties, we hypothesize that fermenting peach beverage using both peel and pulp could enhance the nutrition and flavor of a peach-based beverage. Thus, three different raw methods, namely crushing puree with peel, crushing puree without peel and squeezing juice with peel, were applied in this research. The nutritional, aromatic and sensory properties of different fermented peach purees and juices were evaluated by analyzing the changes in sugars, organic acids, anthocyanins, polyphenols, carotenoids and volatile compounds before and after fermentation.

## 2. Materials and Methods

### 2.1. Strain, Chemicals and Reagents

The strain was purchased from China Center of Industrial Cultural Collection (Beijing, China). Sugars and organic acid standards (>99%) were obtained from Dr. Ehrenstorfer GmbH Co. (Augsburg, Bavaria, Germany). Other chemical standards, including carotenoids, polyphenols, Vitamin C and 2-octanol, were purchased from Sigma-Aldrich Co. (St. Louis, MO, USA). Folin–Ciocalteu reagent and 1,1-diphenyl-2-picrylhydrazyl (DPPH) were obtained from Sigma-Aldrich. Methanol, acetonitrile and acetic acid (>99.7%) were obtained from Dikma Tech. Inc. (Foothill Ranch, CA, USA). EDTA-Na_2_-Ca, methyl tert-butyl ether (MTBE), butylated hydroxytoluene (BHT), triethanolamine (TEA) and other analytic chemicals were obtained from Shanghai Macklin Biochemical Co. (Shanghai, China). Rogosa and Sharpe (MRS) was obtained from HopeBio-Tech Co. (Qingdao, China). Water was purified with Milli-Q Academic (Millipore, Guyancourt, France).

### 2.2. Peach Material and Sample Preparation

Fresh, intact and matured (12° Brix) peaches (*Prunus persica* Batsch cy ‘huangjinmi NO.5’) were harvested from the Xinxiang base of Zhengzhou Fruit Research Institute (Xinxiang, China, 113.78° E, 35.13° N). After washing, the peaches were randomly divided into three groups and subjected to three different pretreatment methods: crushing into puree with peel (CWP), crushing into puree without peel (COP) and squeezing into juice with peel (SWP). Prior to these treatments, all peaches were cut into pieces and blanched in boiling water for 30 s. The CWP and COP samples were prepared using a blender (JYL-G12E, Joyoung, China), and SWP samples were processed using a juicer (MJ-JS20A2, Midea, Foshan, China) and then centrifuged at 3220× *g* for 15 min (5810 R, Eppendorf, Hamburg, Germany). Each pretreatment was sterilized at 121 °C for 15 min (DSX-280B, Shenan, China) and cooled to room temperature (26 °C) before inoculation.

### 2.3. Fermentation Process

The strain was reactivated in MRS broth (37 °C, 48 h) by an incubator (DPH-9052, Pudong Rong-Feng, Shanghai, China). The obtained cultures were then inoculated (5%, *v*/*v*) into pasteurized samples and incubated (37 °C, 48 h) until reaching the exponential growth phase. These cultures were subsequently used as starters for peach sample (400 mL) fermentation (FPs) at 37 °C for 60 h. A sterilized sample without inoculation was used as the unfermented peach sample (UPs).

### 2.4. Viable Cell and pH Measurement

The total count of the viable cells was determined by the standard serial dilution method [17] and the results were reported as colony-forming units per milliliter (CFU/mL) of sample. The pH was measured at room temperature (26 °C) using a digital pH meter (PHS-3C, Inesa, Shanghai, China).

### 2.5. Determination of Total Phenol Content and Antioxidant Activity

Peach samples were centrifuged at 3220× *g* for 15 min. The supernatant was stored at −20 °C before analysis.

The total phenol content (TPC) in peach samples was determined using the Folin–Ciocalteu method [18] with slight modifications. Briefly, 0.5 mL of sample was mixed with 2.5 mL of Folin–Ciocalteu reagent in a tube and incubated at 50 °C for 5 min (HH-W600, Putian, Jintan, China). After incubation, 2 mL of Na_2_CO_3_ (75 g/L) was added, and the mixture was allowed to react in the dark for 30 min. The absorbance was then determined at 760 nm using a UV spectrophotometer (Specord 50, Analytic Jena, Jena, Germany). The TPC was expressed as gallic acid equivalents (μg GAE/mL).

Antioxidant activity was assessed by measuring the DPPH radical scavenging capacity [19]. A volume of 0.2 mL of samples or vitamin C (VC) standards was mixed with 4.8 mL of 0.1 mM DPPH solution and left in the dark for 30 min. The absorbance was then recorded at 517 nm using a UV spectrophotometer. Results were expressed as VC equivalents (μg VC/mL).

### 2.6. Determination of Sugars and Organic Acids by HPLC

Sugars and acids were analyzed using a high-performance liquid chromatography (HPLC) system (e2695, Waters Corp., Wilford, MA, USA) according to the method previously reported by Li et al. [20] with some modifications. Sample pretreatment was conducted as outlined in Section 2.5. All solutions and samples were filtered through a 0.22 μm membrane filter.

Sugars were separated by a Waters Sugar-Pack I column (6.5 × 300 mm, 10 μm) paired with a 2414 refractive index detector (Waters, Wilford, MA, USA). The mobile phase was 50 mg/L EDTA-Na_2_-Ca solution, and the column temperature was maintained at 80 °C. The flow rate and injection volume were 0.5 mL/min and 10 μL. Organic acids were chromatographed at 35 °C on a Waters X select^®^ HSS T3 column (4.6 × 250 mm, 5 μm) equipped with a UV-2489 detector (Waters, Wilford, MA, USA), and were detected at 210 nm. The mobile phase was (NH_4_)_2_HPO_4_ (0.02 M, pH 2.4) and the flow rate was 1 mL/min. The injection volume was 10 μL. Sugars and organic acids were identified and quantified by comparing relative retention time and peak area of standards.

### 2.7. Determination of Polyphenols by HPLC

Polyphenols were extracted using a slightly modified version of the method described by Wu et al. [12]. A 20 mL peach sample was extracted three times with ethyl acetate (1:1, *v*/*v*). The upper layer was collected and concentrated using a rotary evaporator (RE-52AA, Yarong, Shanghai, China). The residue obtained was then dissolved in 2 mL of methanol for further analysis. Polyphenols in the peach sample were identified using an e2695 HPLC system equipped with a UV-2489 detector. Separation was carried out on a Waters Symmetry C18 column (4.6 × 250 mm, 5 μm) maintained at 35 °C. The mobile phase consisted of two solvents: (A) 0.2% acetic acid aqueous solution (*v*/*v*) and (B) acetonitrile. The gradient program was set as follows: 0 min, 95%A; 8 min, 88.5%A; 10 min, 88%A; 22 min, 83%A; 23 min, 81%A; 28 min, 80%A; 38 min, 73%A; 44 min, 70%A; 47 min, 67.5%A; 52 min, 15%A; 57 min, 100%A; 65 min, 5%A, at a flow rate of 1 mL/min. The injection volume was 10 μL, and detection was performed at 280 nm. Concentrations were reported in μg/mL.

### 2.8. Determination of Carotenoids by HPLC

Carotenoid compounds were analyzed according to previous research with minor adjustments [21]. A 20 mL peach sample was freeze-dried at −80 °C, 0.09 mbar for 48 h (Alpha 2-4 LSCplus, Christ, Osterode, Germany). The dried samples were extracted three times (30 min per time in the dark) with 20 mL extraction solvent (n-hexane: acetone: ethyl alcohol at a 2:1:1 ratio). The mixture was subsequently centrifuged at 3220× *g* (4 °C) for 10 min. The supernatant was collected and washed with a 10% (*w*/*v*) aqueous NaCl solution until a neutral pH was achieved. Then, the extract was concentrated at 26 °C (Concentrator plus, Eppendorf, Germany) and dissolved in 2 mL of MTBE. Then, 2 mL of 10% (*w*/*v*) KOH in methanol was added, and the mixture was saponified in the dark for 10 h. After saponification, the mixture was washed again with aqueous NaCl solution to neutrality. The upper layer was collected, and concentrated at 26 °C. Finally, it was dissolved in 500 μL of MTBE and filtered through a 0.22 μm microfilter before analysis.

Carotenoids were determined by an e2695 HPLC system equipped with a 2489 UV-detector. Separation was performed on a YMC C30 column (4.6 × 250 mm, 5 μm) (Wilmington, NC, USA) at 30 °C, with an injection volume of 10 μL. The flow rate was set at 1 mL/min, and the detection was carried out at 450 nm. Solvent A consisted of a mixture of acetonitrile and methanol (3:1, *v*/*v*) containing 0.01% (*w*/*v*) BHT and 0.05% (*w*/*v*) TEA. Solvent B was MTBE containing 0.01% (*w*/*v*) BHT. The gradient program was as follows: 0–15 min, 98–97%A; 15–25 min, 94%A; 25–38 min, 86%A; 38–43 min, 75%A; 43–47 min, 50% A; 47–50 min, 26%A; 50–53 min, 98%A; 53–55 min, 98%A. Carotenoids were identified and quantified by comparing their relative retention times and peak areas of standards and results were expressed as μg/100 mL.

### 2.9. Determination of Volatile Organic Compounds by GC-MS

The volatile organic compounds (VOCs) present in the peach sample were determined using headspace solid-phase microextraction (HS-SPME) combined with gas chromatography–mass spectrometry (7890A-5975C GC-MS, Agilent, Santa Clara, CA, USA) following the procedure described by Wang et al. [22]. In brief, 7 mL of the peach sample, along with 2 g of NaCl and 10 μL of 2-octanol (used as an internal standard at 20 μg/L), were placed into a 25 mL headspace vial. The mixture was heated at 40 °C for 30 min on a magnetic stirring device (PC-420D, Corning, NY, USA) with stirring at 500 rpm to reach equilibration. Then, VOCs were extracted using an SPFE fiber (DVB/CAR/PDMS 50/30 μm, Supelco, Bellefont, PA, USA) at 40 °C for 30 min. Subsequently, the fiber was retracted and inserted into the GC injection port set at 250 °C for desorption over 8 min. The chromatographic conditions were as follows: helium served as the carrier gas at a flow rate of 1.0 mL/min. The column (DB-225ms, 30 m × 0.25 mm × 0.25 μm, Agilent, USA) temperature was initially held at 40 °C for 3 min, then ramped up to 160 °C at a rate of 3 °C/min and held for 2 min, followed by an increase to 220 °C at a rate of 8 °C/min, where it was maintained for 3 min. The temperature of transfer line, ion trap and quadrupole were 280 °C, 230 °C and 150 °C, respectively. MS spectrometry was performed using electron impact ionization at 70 eV at 1 s/scan, operating in full scan mode over a mass-to-charge (m/z) range of 50–550. The identification of VOCs was achieved by matching their mass spectral against the NIST 1.1 commercial mass spectral library. The relative concentrations of volatile compounds were calculated using the following equation:

(1) $$Concentration\left(\frac{\mu g}{L}\right)=\frac{Peak\;\;of\;compound}{peak\;of\;internal\;standard}\times concentration\;of\;internal\;standard$$

### 2.10. Odor Activity Values (OAVs)

The contribution of each volatile compound to the aroma of the peach sample was assessed qualitatively using its related descriptor and quantitatively through its OAV. OAVs were calculated by dividing the measured concentration of the volatile compound with its odor threshold.

### 2.11. Sensory Evaluation

Sensory evaluation was conducted based on the method described by Liu et al. [23] with some modifications. The evaluation panel consisted of 20 food science-related scholars (10 women and 10 men between 20 and 40 years) from Zhengzhou Fruit Research Institute. The sensory evaluation of each set of peach sample contained six samples, which were presented to the panelists in a random order using coded plastic disposable cups at 25 °C. Mouthwash was provided between evaluations. The evaluation criteria included color, mouthfeel, odor, consistency and overall acceptance. Panelists rated each attribute on a 10-point scale, and the average scores for each characteristic were calculated and displayed in a spider diagram.

### 2.12. Statistical Analysis

Experiments were performed in triplicate and results were expressed as mean ± standard deviation. The statistical analysis was performed by variance analysis using GraphPad Prism 8 (GraphPad Software Inc., San Diego, CA, USA). Analysis of variance (ANOVA) was used to assess the significance of the treatment effects (*p* < 0.05). Heatmap and clustering analysis of GC-MS results was visualized by TBtools V 2.01. The differential metabolites screening of volatiles was performed using the Metware Cloud, a free online platform for data analysis (https://cloud.metware.cn/), and a horizontal lollipop was plotted by https://www.bioinformatics.com.cn (last accessed on 25 August 2025), an online platform for data analysis and visualization.

## 3. Results and Discussion

### 3.1. Viable Counts, pH Values, Total Phenol and Antioxidant Activity

The viable bacteria number, pH, total content of phenol and antioxidant activity of peach purees and juice from different pretreatments are shown in Table 1. As expected, the viable bacterial count of all fermented peach samples exceeded 9.0 log CFU/mL, and there was no significant difference among different pretreatments. However, the pretreatments significantly affected the pH of unfermented samples, the peach puree crushing with peel (CWP) showed the lowest pH value, and the peach juice squeezing with peels (SWP) had the highest pH value. This may be due to the dissolution of organic acids contained in the peach sample during processing. Moreover, *lactobacillus* fermentation was processed with a significant (*p* < 0.05) decrease in the pH of the samples due to the accumulation of lactic acid, and there was a significant difference within each fermented group. Compared with the unfermented group, the pH reduced by 0.99 (CWP), 0.97 (COP) and 0.99 (SWP), respectively. It confirmed that using peach fruit as a fermentation substrate could effectively promote the growth and metabolism of *Lactiplantibacillus plantarum* 21802. The different pretreatments did not affect the growth of this strain, but would affect its acid-producing capacity.

Among the unfermented samples, CWP exhibited the highest total phenol content and antioxidant activity. COP showed the lowest phenol content, but its antioxidant activity was higher than that of SWP. It indicates that the peach peel is rich in phenolic compounds, and the different polyphenol compositions could lead to different antioxidant activities. Similarly, fermented CWP also showed the highest total phenolic content and antioxidant activity, which increased by 23.09 μg/mL (18.91%) and 25.13 μg VC/mL (35.36%), respectively. COP and SWP increased by 19.02 μg/mL (18.18%) and 28.27 μg VC/mL (46.24%), 8.59 μg/mL (7.34%) and 17.52 μg VC/mL (29.62%), respectively. It means that the polyphenols in the CWP group were more likely to be converted into polyphenols with high antioxidant activity during the fermentation process.

### 3.2. Sugars and Organic Acids

The effects of fermentation on the sugars and acids of peach samples from different pretreatments are listed in Table 2. The total sugar content in unfermented samples ranged from 67.56 to 70.79 mg/mL, in which COP had the lowest total sugar content. Sucrose was the major sugar component in all unfermented samples, which accounts for 69.29% (CWP), 69.38% (COP) and 70.31% (SWP), respectively. There was also no significant difference among different samples. However, the content of glucose and fructose in SWP were significantly lower than other unfermented samples, and COP showed the lowest sorbitol concentration. After fermentation, the total sugars decreased by 3.15 mg/mL (CWP), 3.06 mg/mL (COP) and 2.58 mg/mL (SWP), respectively. Among them, the content of glucose and fructose in all samples decreased significantly, while the contents of sucrose and sorbitol had no changes. The amount of glucose and fructose consumption during fermentation in the three groups were 2.06 and 0.87 mg/mL (CWP), 2.21 and 0.86 mg/mL (COP) and 2.21 and 0.33 mg/mL (SWP), respectively. The results indicated that only glucose and fructose could be metabolized during Lactobacillus fermentation, which is in agreement with the findings in our previous study [11]. Moreover, the sugar metabolism of *Lactobacillus* were various in peach purees and juices, and similar results have been previously reported in different carrot juices and organ juices [24,25]. The highest sugar consumption in the CWP group suggested more efficient utilization of *Lactobacillus* in this group, and confirms that peach puree is a better matrix for lactic acid fermentation.

A total of six organic acids were detected in the samples, and there was no significant difference in total acid content among different pretreatments. Malic acid was the largest organic acid in unfermented samples, which accounts for 53.88% (CWP), 56.29% (COP) and 55.27% (SWP), respectively. The malic acid content in unfermented samples ranged from 2.22 to 2.37 mg/mL, which was close to the average malic content (2.62 mg/mL) of peach juice from 12 different peach cultivars determined by Li et al. [20]. *Lactobacillus* fermentation resulted in a negative effect on malic acid, which was consumed completely in all samples, as was fumaric acid. Similarly, the content of citric acid decreased by 15.22% (CWP), 20.65% (COP) and 24.47% (SWP), respectively. Conversely, lactic acid is considered as an indicator of *Lactobacillus* fermentation [26], increasing by 8.60 mg/mL (CWP), 7.55 mg/mL (COP) and 8.17 mg/mL (SWP), respectively. In addition, the accumulation of quininic acid was detected as well, which increased by 57.43% (CWP), 32.97% (COP) and 60.42% (SWP). In this study, malic acid was the major organic acid carbon source during peach sample fermentation, but *Lactiplantibacillus plantarum* 21802 would compensate for insufficient malic acid by increasing the consumption of citric acid. Several studies have proved that malic acid or citric acid could participate in malolactic fermentation or the tricarboxylic acid cycle and generate lactic acid or other products, which depends on the strains and substrate [27,28]. The higher yield of lactic acid indicates a higher degree of glycolytic metabolism and malic–lactic conversion in the CWP group [29]. Similarly, the total acid content in this group was also the highest.

### 3.3. Polyphenols

In order to further investigate the effects of different pretreatment methods on the polyphenols of lactobacilli-fermented peach beverages, the individual polyphenols contained in the sample were analyzed by HPLC. However, due to the trace concentration of some polyphenols, only 11 main polyphenols were detected, which included 4 phenolic acids, 4 flavonols and 3 flavan-3-ols. The changes in polyphenols in different groups are listed in Table 3. The total phenol contents in unfermented peach samples were 27.18 μg/mL (CWP), 28.11 μg/mL (COP) and 27.15 μg/mL (SWP), respectively, and there was no significant difference among different samples. After fermentation, the total phenol content significantly increased by 43.60% (CWP), 28.89% (COP) and 49.36% (SWP), respectively. However, the total phenols determined by HPLC in each group were significantly lower than those obtained by the Folin–Ciocalteu method (TPC, Table 1). This might be caused by the different principles of the measurement method, and the total phenol obtained by HPLC only represents the limited sum of specifically identified polyphenols rather than all polyphenols contained in the sample. However, the trends of total phenol determined by these two methods were similar.

Among them, flavan-3-ols showed the highest content in unfermented CWP and SWP groups, which account for 40.43% and 41.10% of total phenol. However, there was no significant difference between them. Epicatechin, ranging from 49.50% (CWP) to 53.94% (SWP) of total flavan-3-ols, was predominately observed, followed by catechin (34.95–39.59%) and proanthocyanidin B1 (10.23–12.65%). After fermentation, the total flavan-3-ols of peach samples significantly increased by 60.78% (CWP), 46.34% (COP) and 49.91% (SWP), respectively. Unlike the unfermented samples, the total amount of flavan-3-ols was significantly higher in the CWP group (17.67 μg/mL) than in other fermented samples. All flavan-3-ols increased significantly in all groups, while epicatechin showed the highest concentration in the CWP group (raised by 65.26%). For catechin and proanthocyanidin B1, a significant increase was observed in all samples, but there was no significant difference among the samples. The most interesting discovery is that the proanthocyanidin B1 content was 1.54–2.31 times higher after fermentation.

The total phenolic acid content in each unfermented group was similar; only p-coumaric acid had a higher concentration in the CWP group. Protocatechuic acid was the major phenolic acid in the peach samples, which accounts for 92.25% (CWP), 92.54% (COP) and 91.24% (SWP) of the total phenolic acid. The contents of chlorogenic acid, vanillic acid and p-coumaric acid were very low in all unfermented samples. *Lactobacillus* fermentation promoted the degradation of protocatechuic acid, and decreased by 62.04% (CWP), 60.58% (COP) and 35.89% (SWP), respectively. Among them, SWP showed the lowest concentration after fermentation. Conversely, the levels of chlorogenic acid in the peach samples were significantly increased, and the increase amounts in each group were 7.24 μg/mL (CWP), 6.53 μg/mL (COP) and 6.76 μg/mL (SWP), respectively. Consequently, it became the main phenolic acid in the peach samples after fermentation, accounting for 59.20% (CWP), 56.55% (COP) and 50.53% (SWP) of total phenolic acids. Higher concentrations of chlorogenic acid were detected in the fermented CWP (7.59 μg/mL) and SWP (7.13 μg/mL) groups. In addition, a significant accumulation of vanillic acid in the peach samples were observed after fermentation, which increased 2.27–2.68 times. The total phenolic acids increased after fermentation, while only the SWP group showed a significant difference.

Flavonols only accounted for 20.16% (CWP), 22.48% (COP) and 23.57% (SWP) of total phenol in the unfermented peach samples. Among them, isohamnetin-3-O-glucoside and quercetin-3-O-glucoside, accounting for 44.16% and 50.00% (CWP), 43.35% and 52.37% (COP) and 45.00% and 51.25% (SWP) of total flavonols, were predominantly presented in the peach samples. It is interesting that all fermentation groups showed a significant decrease in isorhamnetin-3-O-glucoside and an increase in isorhamnetin. And the final amount of total flavonols increased by 55.84% (CWP), 33.07% (COP) and 51.72% (SWP), respectively.

In conclusion, the present results indicated that the composition of polyphenols and their metabolism during *Lactobacillus* fermentation were highly varied among different pretreatment groups. The same finding was presented in pomegranate juice [30], which suggested that it would be caused by the different distribution of bound phenolics in different parts of the fruit. In this study, most polyphenols detected in the samples were in accordance with previous reports [31,32,33], and the level of total phenol in the fermented groups was significantly (*p* < 0.05) higher than in the unfermented groups, especially in the CWP and SWP groups. It indicated that the production of polyphenols was more rapid than their consumption during CWP and SWP group fermentation, which suggested that these two groups were more conducive for polyphenol accumulation. The previous literature confirmed that polyphenols exhibited higher concentration in the peel compared to the pulp, especially bounded polyphenols [31,34]. The maceration of peel would facilitate the entry and retention of polyphenols from the peel into the peach sample [35], and the soluble conjugated or insoluble bounded polyphenols contained in the samples would be released by enzymatic metabolism during *Lactobacillus* fermentation [36,37], resulting in the increase of polyphenols in these two groups. Compared to other polyphenols, the accumulation flavan-3-ols were more prominent in these two fermentation groups, especially epicatechin. Several studies suggested that epicatechin would not be catabolized by *Lactobacillus* [38], and is mainly released from bounded flavan-3-ols in peel. Similar to epicatechin, catechin and chlorogenic acid are also mainly derived from the release of bound phenols, while quercetin and isorhamnetin are related to the enzymatic hydrolysis of β-glucosidase [27].

### 3.4. Carotenoids

Changes in the three carotenoids in different pretreatments are shown in Table 4. The total carotenoid content in unfermented peach samples ranged from 86.33 μg/100 mL (SWP) to 2008.44 μg/100 mL (CWP), among which β-carotene was the dominant carotenoid in the peach samples, accounting for 75.92% (CWP), 73.94% (COP) and 100% (SWP) of total carotenoids, respectively. Zeaxanthin and β-cryptoxanthin were not detected in the SWP groups. Moreover, the content of these two compounds in the CWP group were significantly higher than those in the COP group. It indicates that different raw material methods have a significant impact on the content of carotenoids in peach purees and juice.

After fermentation, the highest level of β-carotene was detected in the COP group, as well as zeaxanthin and β-cryptoxanthin. Their contents increased by 4.80%, 17.54% and 8.88%, respectively. Conversely, the levels of these carotenoids decreased in the CWP group, which reduced by 26.82%, 35.38% and 32.60%, respectively. The SWP group showed the lowest total carotenoid content, which was only 5.05% of the COP group after fermentation. Statistically, the total carotenoid content increased by 7.57% in the COP group, decreased by 28.09% in the CWP group and there were no changes in the SWP group.

β-carotene, β-cryptoxanthin and zeaxanthin were commonly presented in peaches [39,40]. These compounds are lipid soluble and associated closely with proteins in chromoplasts. The double membrane of the chromoplasts, along with the layers of the cell membrane and cell wall, enable them to be stably present in the chromoplasts of plants cells [41]. The greatest amount of carotenoid would remain in the pomace fraction rather than transfer into the juice [41,42], and that is the reason for the low carotenoid concentration in the SWP group. Moreover, the low concentrations of zeaxanthin and β-cryptoxanthin present in the peaches result in an unquantified amount being transferred to the juice during processing. Several reports suggested that *Lactobacillus* fermentation would enhance carotenoid release and improve their extraction [43,44,45]. Simultaneously, carotenoids are also easily oxidated by heating, oxygen and enzymes during fermentation [43,46], and the random carbon–carbon bond cleavage is regarded as the main mechanism for this reaction [47,48]. However, the degradation of carotenoids was significant different among different pretreatment samples, with the sample produced without peels (COP) - showed the lowest carotenoid degradation. The higher degree of carotenoid degradation in CWP and SWP might be caused by the oxidation of polyphenols contained in the peels [49]. Despite this, CWP also had a high level of carotenoids because of its high initial content.

### 3.5. VOCs

**Figure 1:**
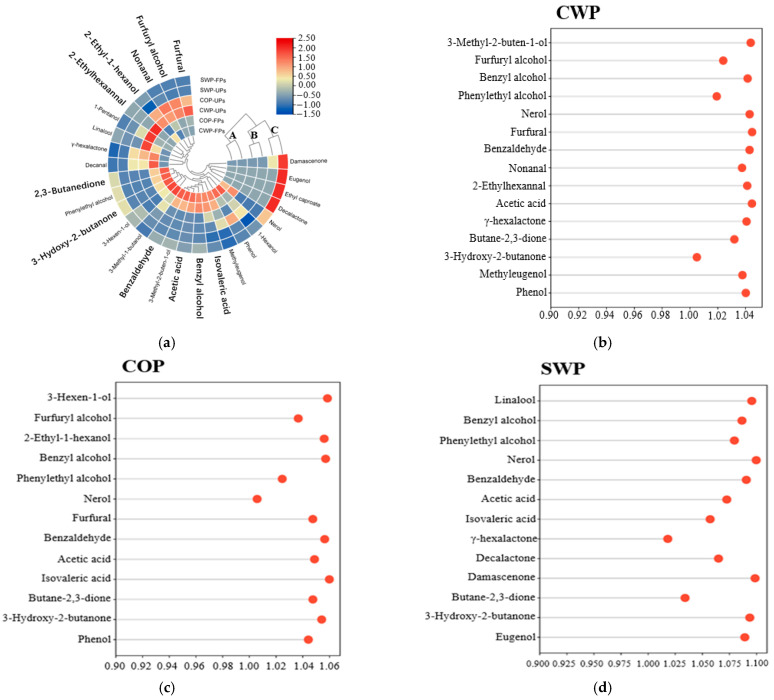
GC-MS results of unfermented and fermented peach samples from different raw material methods; (**a**) Heatmap visualization of all samples; (**b**) Analysis of differential metabolites before and after fermentation in the CWP group; (**c**) Analysis of differential metabolites before and after fermentation in the COP group; (**d**) Analysis of differential metabolites before and after fermentation in the SWP group.

To investigate the effects of fermentation on the aroma profiles of each pretreatment, the composition and relative content of aroma compounds in the samples were analyzed and identified using HS-SPME coupled with GC-MS. A total of 27 volatile organic compounds were identified in unfermented and fermented peach samples from three different pretreatments, which included 11 alcohols, 5 aldehydes, 2 acids, 3 esters, 3 ketones and 3 phenols (Table S1). Based on the different composition and concentration of VOCs in different samples, the hierarchical cluster analysis divided all samples into three clusters by a similarity criterion, namely A, B and C (Figure 1a). Cluster A was characterized by the fermented CWP and COP groups, and Cluster B included the unfermented CWP and COP samples, demonstrating that *Lactobacillus* fermentation had an obvious influence on the VOC profile in these two pretreatment groups. However, unfermented and fermented SWPs were clustered in Cluster C, which indicated that the composition and concentration of volatiles in these two groups were not significantly different. In Figure 1b–d, the differential metabolites before and after fermentation in each group were screened according to the criteria of VIP > 1 and |*p* (error)| > 0.5.

The total VOC contents in each unfermented sample were 680.02 μg/L (CWP), 424.62 μg/L (COP) and 66.77 μg/L (SWP) (Table S2). Among them, peach puree obtained by crushing with peel (CWP) had the richest volatile compounds, which were 1.60-fold and 10.18-fold of the COP and SWP groups. It indicates that the peel and pulp of peach fruit contained more volatiles. Most of the volatile organic compounds significantly increased after fermentation, resulting in the final total volatile organic compounds increasing by 1599.86 μg/L (CWP), 1396.99 μg/L (COP) and 378.11 μg/L (SWP), respectively. Compared with the CWP and COP groups, the fermentative biotransformation of volatiles was significantly limited in the juice matrix, although the total volatiles increased after fermentation. It indicated that *Lactobacillus* fermentation with peel and pulp were more conducive to the release of volatiles. The presence of peel and pulp could provide more aroma precursor substances by *Lactobacillus* metabolism, such as citric acid cycle, glycolysis, esterification and enzymatic reaction [11,50,51]. Compared with the CWP group, COP showed lower volatiles concentration, which might be caused by peeling [35].

**Figure 2:**
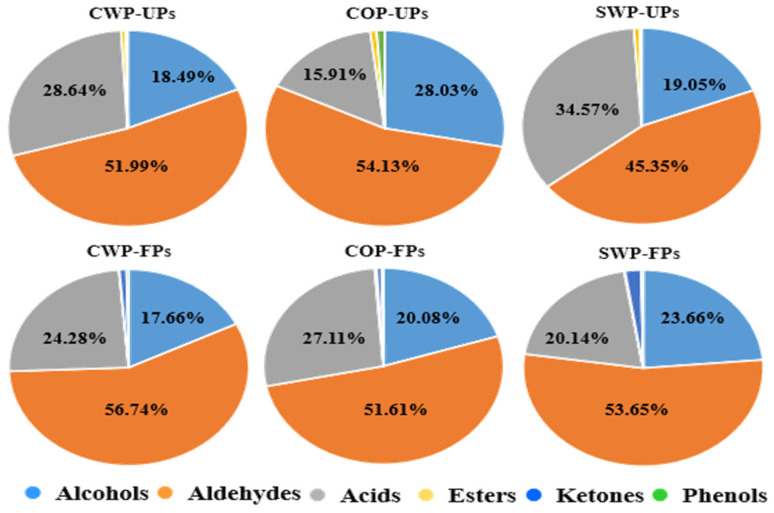
The proportion of different kinds of VOCs in unfermented and fermented peach samples from different raw material methods.

Aldehydes were the most abundant volatile organic compounds in the unfermented groups, accounting for 51.99% in the CWP group, 54.13% in COP and 45.35% in SWP, respectively. After fermentation, the total aldehydes increased by 940.04 μg/L (CWP), 710.37 μg/L (COP) and 208.38 μg/L (SWP) and accounted for 56.74%, 51.61% and 53.65% of total VOCs, respectively (Figure 2). It is interesting to note that the proportion of aldehyde only decreased in the COP fermentation group, indicating a lower level of substrates for aldehyde conversion in this group. However, the common phenomenon among different pretreatments was that the content of benzaldehyde significantly increased after fermentation, by 1180.88 μg/L (CWP), 814.7 μg/L (COP) and 213.57 μg/L (SWP), respectively (Table S1). This result is consistent with our previous studies, and its increase was caused by amino acid metabolism and fatty acid oxidation [11,52]. Including benzaldehyde, there was a total of four aldehydes with VIP values exceeding 1 in the CWP group (Figure 1b). Furfural, the most abundant volatile compound in the unfermented sample, was significantly reduced (81.43%) in this group, as well as in the COP group. Additionally, the concentration of nonanal decreased by 84.91%, and 2-ethylhexaannal was completely consumed during fermentation. The same results were also found in jujube juice fermentation [53], and their decrease might be caused by their reduction to alcohols or oxidation to acids [54].

Notably, Isovaleric acid was the only volatile acid detected in the unfermented peach sample. Following fermentation, the total acid content significantly increased by 358.92 μg/L (CWP), 426.21 μg/L (COP) and 66.52 μg/L (SWP), respectively. Among them, acetic acid emerged as the main differentiating metabolite of volatiles in all pretreatment groups, showing a marked elevation of 190.04 μg/L (CWP), 151.80 μg/L (COP) and 15.99 μg/L (SWP), respectively. Moreover, the concentration of isovaleric acid also showed a significant increase of 168.88 μg/L (CWP), 274.41 μg/L (COP) and 50.53 μg/L (SWP) (Table S1). The generation of isovaleric acid mainly originates from leucine catabolism [55], and this result may be related to the different leucine content in the sample after different pretreatments.

Statistically, the proportion of alcohols within the total VOC contents in unfermented peach samples was 18.49% for CWP, 28.03% for COP and 19.05% for SWP (Figure 2). After fermentation, the total alcohol content significantly increased by 276.95 μg/L (CWP), 246.71 μg/L (COP) and 92.52 μg/L (SWP), representing 17.66%, 20.08% and 23.66% of total VOCs, respectively (Figure 1, Table S2). Furfuryl alcohol was the predominant alcohol in the unfermented CWP and COP groups (VIP > 1), accounting for 56.37% and 59.51% of total alcohols, respectively. However, its concentration sharply decreased by 54.65 μg/L and 55.07 μg/L after fermentation, making up only 4.04% and 4.31% of total VOCs, respectively. Similarly, 2-ethyl-1-hexanol constituted a significant portion of total alcohols (33.29% in CWP-UFs and 32.27% in COP-UFs). Notably, the VIP value of the CWP treatment group was below 1, indicating that the reduction in 2-ethyl-1-hexanol during fermentation was not significant in this group, whereas it decreased markedly by 23.23 μg/L in the COP group. In contrast, benzyl alcohol, phenylethyl alcohol and nerol were observed as new alcohols in fermented peach samples, with VIP values exceeding 1 across all pretreatment groups. Among these, benzyl alcohol was the primary alcohol, increasing by 336.76 μg/L (CWP), 320.16 μg/L (COP) and 80.04 μg/L (SWP), respectively. Benzy alcohol is produced through the degradation of phenylalanine, and is typically formed during fermentation [56,57]. Additionally, 3-methyl-2-buten-1-ol appeared as a new compound after fermentation, although its VIP value was greater than 1 only in the CWP treatment group.

Furthermore, three esters, three ketones and three phenols were detected in the unfermented peach samples. These compounds were present in very low amounts, accounting for 0.87% (CWP), 1.93% (COP) and 1.03% (SWP) of the total VOCs, respectively. After fermentation, their total contents increased by 23.95 μg/L, 13.69 μg/L and 10.68 μg/L, respectively. Interestingly, the content of ketones significantly increased after fermentation, contributing to 89.52%, 85.61% and 86.61% of their total increase. Two new ketones, namely 2,3- butanedione and 3-hydroxy-2-butanone, were detected in all fermentation groups (VIP > 1), and the highest concentrations were shown in the CWP fermented peach samples. Similar results were found in kiwi juice [53]. These two volatiles are mainly produced in the citric acid cycle and glycolysis process, in which 2,3-butanedione is synthesized through the combination of oxalacetate decarboxylase, α-acetolactate and oxidative decarboxylation, then converted to 3-hydroxy-2-butanone under the action of diacetyl reductase [58]. Regarding phenols, CWP and COP groups presented higher total phenol concentrations after fermentation, increasing by 5.73 μg/L and 0.19 μg/L, respectively. Among them, methyleugenol was the dominant phenol in these two groups after fermentation, accounting for 64.88% and 84.19%, but it was absent in the SWP fermented group. Among esters, γ-hexalactone, a characteristic aromatic ester in unfermented peach samples, decreased after fermentation.

### 3.6. Analysis of the OAVs

**Figure 3:**
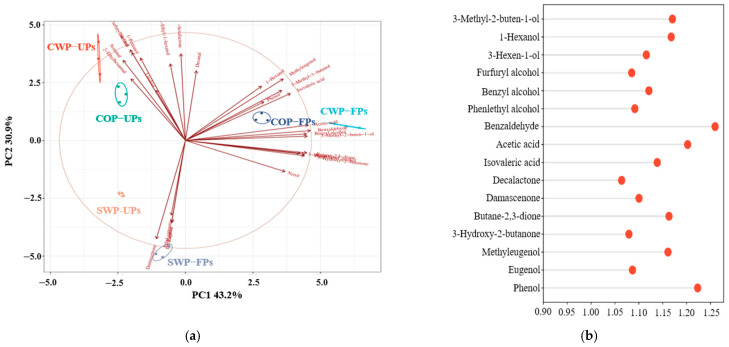
Analysis of the differences in the composition of VOCs among different fermentation groups; (**a**) PCA analysis of peach samples from different raw material methods; (**b**) Analysis of differential metabolites in fermented peach samples from different groups.

*Lactobacillus* fermentation significantly influenced the aroma of peach samples. The PCA results showed a clear classification trend among the samples, with a total of 16 aroma compounds exhibiting VIP values greater than 1 detected among different fermentation groups (Figure 3). In order to clarify the flavor characteristics of the various fermentation products, the OAVs were calculated for each sample, and nine key aroma compounds (OAVs > 1) are listed in Table 5. Combining the analysis of differential aroma metabolites, there were five key aroma compounds which were identified as the main differential metabolites, including three alcohols (3-methyl-2-buten-1-ol, furfuryl alcohol and benzyl alcohol), one aldehyde (benzaldehyde) and one ketone (2,3-butanedione).

Compared with the unfermented group, the OAVs of aroma substances changed significantly after fermentation. The primary aroma-contributing alcohols shifted from furfuryl alcohol to benzyl alcohol, indicating that *Lactobacillus* fermentation reduces the bread-like aroma [61], while enhancing floral and rose notes [63]. Similarly, the OAVs of nonanal decreased, whereas benzaldehyde increased after fermentation, indicating the green aroma [53] in fermented peach samples was diminished, while the almond flavor [64] became more prominent. Meanwhile, the OAVs of ketones also increased in all fermented samples, indicating that the fermented peach samples exhibited a stronger, pleasant, sweet and buttery aroma [61] compared to the unfermented samples.

Moreover, the flavor profiles of each fermentation sample were various. Among them, the CWP-FPs showed the highest OAV levels for most compounds (3-methyl-butanol, benzyl alcohol, benzaldehyde and 2,3-butanedione), indicating that this group had the strongest flavor compared to the others. The OAVs of these VOCs in the COP-FPs were lower than those in the CWP-FPs. However, only the OAV of 3-methyl-1-butanol was below 1, which indicated that its aroma was relatively weak and lacked the fruity aroma compared with the CWP fermentation group. For the SWP fermentation group, only the OAV of 2,3-butanedione was greater than 1, indicating that this group only has a distinct buttery aroma, without any notable fruity, floral or sweet scents. Based on the comprehensive analysis results of volatiles composition and odor profiles, *Lactobacillus* fermentation could promote the formation of positive flavor compounds in peach samples, especially when fermented with both peel and pulp.

### 3.7. Sensory Evaluation

**Figure 4:**
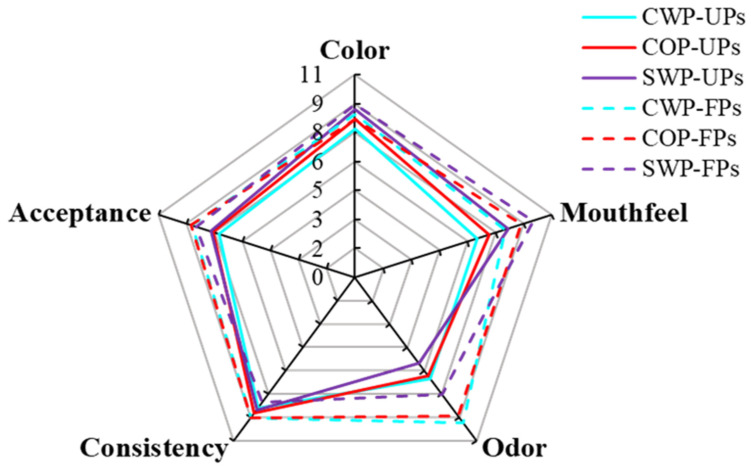
Sensory evaluation radar chart of unfermented and fermented peach samples from different raw material methods.

The sensory evaluation of odor, color, mouthfeel, consistency and overall acceptance for each sample is shown in Figure 4. It was found that *Lactobacillus* fermentation significantly influenced the sensory characteristics of samples, particularly odor and mouthfeel. The odor satisfaction scores of all fermented groups were higher than those of the unfermented groups. The CWP-FPs received the highest odor score, followed by the COP-FPs and SWP-FPs. This indicates that *Lactobacillus* fermentation with peel and pulp generated more aromatic compounds that were highly appreciated by customers, aligning with the findings from the VOC analysis. However, the presence of peel and pulp was not friendly to mouthfeel. Generally, peach juice without pulp and peel aligns better with consumers’ expectations of traditional juice, as the presence of peel and pulp reduce the refreshing quality of products, especially the peel. The mouthfeel satisfaction of the CWP group before and after fermentation were the lowest, followed by the COP group. The SWP treatment group exhibited drawbacks, such as insufficient flavor and noticeable sedimentation. In contrast, products obtained from CWP and COP fermentation were more homogeneous. Overall, the products from the COP treatment group achieved the highest acceptance after fermentation.

## 4. Conclusions

This study examined the microbial growth, physicochemical properties, volatile composition and sensory profiles of fermented peach purees and juice produced using different raw material preparation methods. Although *Lactobacillus* fermentation positively influenced the conversion and release of phytochemicals in all peach samples, significant differences were observed among the samples in terms of physicochemical composition and sensory profile. The CWP peach puree exhibited the highest sugar consumption, polyphenol content and antioxidant activity after fermentation, along with the greatest biotransformation of chlorogenic acid and epicatechin. In the COP group, *Lactobacillus* showed a preference for converting carotenoids, especially β-carotene. Benzaldehyde was the predominant aroma compound across all fermented samples, while 2,3-butanedione emerged as a characteristic aroma substance after fermentation. The CWP peach puree contained the highest level of VOCs, followed by the COP group. For the SWP group, the polyphenol content was second only to that of the CWP group, but carotenoids and volatile contents were the lowest after fermentation. Moreover, this group had the highest mouthfeel satisfaction. In conclusion, pretreatment without peeling (CWP and SWP) is beneficial for improving the polyphenol concentration, while it had a negative impact on carotenoids conversion during fermentation. Fermentation with both peel and pulp (CWP and COP) promotes the formation of aroma compounds. Based on the current results, the greatest challenge for the CWP group is low mouthfeel satisfaction and carotenoid concentration caused by the presence of peel. Puree crushing without peel would be preferable for peach-based fermented beverages, but this group also had the problem of relatively low mouthfeel satisfaction due to insoluble pulp and particles, and the application of high (ultra-high) pressure homogenization before fermentation could enhance taste and overall quality.

## Figures and Tables

**Table 1:** Changes in viable counts, pH value, total phenols and antioxidant activity of unfermented and fermented peach samples from different raw material methods.

Categories	CWP		COP		SWP	
	UPs	FPs	UPs	FPs	UPs	FPs
Viable counts (log CFU/mL)	ND	9.30 ± 0.19 ^a^	ND	9.31 ± 0.05 ^a^	ND	9.24 ± 0.07 ^a^
pH	4.81 ± 0.00 ^c^	3.82 ± 0.01 ^f^	4.82 ± 0.00 ^b^	3.85 ± 0.00 ^e^	4.85 ± 0.01 ^a^	3.86 ± 0.00 ^d^
Total phenols (μg/mL)	122.11 ± 7.37 ^bc^	145.20 ± 4.21 ^a^	104.61 ± 1.45 ^d^	123.63 ± 6.43 ^bc^	117.11 ± 0.62 ^c^	125.7 ± 0.44 ^b^
Antioxidant activity (μg VC/mL)	71.06 ± 0.10 ^d^	96.19 ± 2.32 ^a^	61.14 ± 0.10 ^e^	89.41 ± 0.83 ^b^	59.15 ± 0.66 ^e^	76.67 ± 2.39 ^c^

Note: Different superscript lowercase letters in the same line represent statistically significant differences (*p* < 0.05). ND means none detected in samples.

**Table 2:** The content of sugars and acids in unfermented and fermented peach samples from different raw material methods (mg/mL).

Categories	CWP		COP		SWP	
	UPs	FPs	UPs	FPs	UPs	FPs
***Sugars***						
Sucrose	49.05 ± 0.35 ^a^	48.75 ± 0.50 ^a^	46.87 ± 0.21 ^b^	46.83 ± 0.17 ^b^	49.15 ± 0.87 ^a^	49.02 ± 1.35 ^a^
Glucose	7.97 ± 0.07 ^a^	5.91 ± 0.14 ^c^	8.15 ± 0.12 ^a^	6.03 ± 0.05 ^c^	7.55 ± 0.07 ^b^	5.34 ± 0.16 ^d^
Fructose	9.43 ± 0.10 ^a^	8.56 ± 0.17 ^b^	9.18 ± 0.35 ^a^	8.32 ± 0.03 ^b^	8.95 ± 0.13 ^b^	8.62 ± 0.26 ^c^
Sorbitol	4.34 ± 0.08 ^ab^	4.41 ± 0.03 ^a^	3.34 ± 0.06 ^c^	3.23 ± 0.02 ^c^	4.26 ± 0.11 ^b^	4.35 ± 0.11 ^ab^
*Total sugars*	70.79 ± 0.58 ^a^	67.64 ± 0.51 ^b^	67.56 ± 0.03 ^b^	64.50 ± 0.11 ^c^	69.90 ± 1.17 ^a^	67.32 ± 1.11 ^c^
***Organic acids***						
Quininic acid	1.01 ± 0.04 ^c^	1.59 ± 0.03 ^a^	0.91 ± 0.04 ^d^	1.21 ± 0.02 ^b^	0.96 ± 0.01 ^cd^	1.54 ± 0.08 ^a^
Malic acid	2.22 ± 0.26 ^a^	ND	2.37 ± 0.06 ^a^	ND	2.36 ± 0.13 ^a^	ND
Shikimic acid	0.01 ± 0.00 ^b^	0.02 ± 0.01 ^a^	0.01 ± 0.00 ^b^	0.01 ± 0.00 ^b^	0.01 ± 0.00 ^b^	0.01 ± 0.00 ^b^
Lactic acid	ND	8.60 ± 0.07 ^a^	ND	7.55 ± 0.08 ^c^	ND	8.17 ± 0.09 ^b^
Citric acid	0.92 ± 0.07 ^a^	0.78 ± 0.02 ^b^	0.92 ± 0.03 ^a^	0.73 ± 0.02 ^bc^	0.94 ± 0.01 ^a^	0.71 ± 0.02 ^c^
Fumaric acid	0.01 ± 0.00 ^a^	ND	0.01 ± 0.00 ^a^	ND	0.01 ± 0.00 ^a^	ND
*Total acids*	4.12 ± 0.15 ^d^	10.99 ± 0.10 ^a^	4.21 ± 0.12 ^d^	9.51 ± 0.08 ^c^	4.27 ± 0.12 ^d^	10.43 ± 0.08 ^b^

Note: Different superscript lowercase letters in the same line represent statistically significant differences (*p* < 0.05). ND means none detected in samples.

**Table 3:** The contents of phenol in unfermented and fermented peach samples from different raw material methods (μg/mL).

Categories	CWP		COP		SWP	
	UPs	FPs	UPs	FPs	UPs	FPs
***Phenolic acid***						
Protocatechuic acid	9.88 ± 0.73 ^ab^	3.75 ± 0.42 ^d^	10.30 ± 0.33 ^a^	4.06 ± 0.82 ^d^	8.75 ± 0.73 ^b^	5.61 ± 0.38 ^c^
Chlorogenic acid	0.35 ± 0.09 ^c^	7.59 ± 0.70 ^a^	0.38 ± 0.02 ^c^	6.91 ± 0.30 ^b^	0.37 ± 0.01 ^c^	7.13 ± 0.07 ^ab^
Vanillic acid	0.34 ± 0.03 ^c^	1.25 ± 0.04 ^a^	0.33 ± 0.01 ^c^	1.08 ± 0.06 ^b^	0.36 ± 0.00 ^c^	1.19 ± 0.06 ^a^
p-coumaric acid	0.15 ± 0.02 ^c^	0.23 ± 0.00 ^a^	0.12 ± 0.02 ^d^	0.17 ± 0.01 ^b^	0.11 ± 0.01 ^d^	0.17 ± 0.01 ^b^
Total phenolic acids	10.71 ± 0.84 ^bc^	12.82 ± 0.89 ^ab^	11.13 ± 0.35 ^bc^	12.22 ± 1.09 ^ab^	9.59 ± 0.74 ^c^	14.11 ± 0.51 ^a^
***Flavonols***						
Isorhamnetin-3-O-glucoside	2.42 ± 0.41 ^b^	1.36 ± 0.14 ^c^	2.74 ± 0.09 ^ab^	1.20 ± 0.05 ^c^	2.88 ± 0.08 ^a^	1.31 ± 0.12 ^c^
Quercetin-3-O-glucoside	2.74 ± 0.50 ^c^	3.93 ± 0.29 ^b^	3.31 ± 0.12 ^c^	3.99 ± 0.31 ^b^	3.28 ± 0.08 ^c^	4.75 ± 0.34 ^a^
Isorhamnetin	0.02 ± 0.00 ^c^	2.12 ± 0.06 ^b^	0.03 ± 0.00 ^c^	2.26 ± 0.10 ^b^	0.03 ± 0.00 ^c^	2.44 ± 0.19 ^a^
Quercetin	0.30 ± 0.10 ^c^	1.14 ± 0.11 ^a^	0.24 ± 0.03 ^c^	0.95 ± 0.06 ^b^	0.20 ± 0.01 ^c^	1.21 ± 0.10 ^a^
Total flavonoids	5.48 ± 1.00 ^b^	8.54 ± 0.55 ^a^	6.32 ± 0.22 ^b^	8.41 ± 0.51 ^a^	6.40 ± 0.15 ^b^	9.71 ± 0.73 ^a^
***Flavan-3-ols***						
Catechin	4.17 ± 0.10 ^b^	5.14 ± 0.10 ^a^	4.22 ± 0.15 ^b^	5.11 ± 0.08 ^a^	3.90 ± 0.01 ^c^	5.10 ± 0.09 ^a^
Epicatechin	5.44 ± 0.63 ^d^	8.99 ± 0.51 ^a^	5.35 ± 0.28 ^d^	6.88 ± 0.52 ^c^	6.02 ± 0.21 ^cd^	7.85 ± 0.59 ^b^
Proanthocyanidin B1	1.39 ± 0.13 ^b^	3.54 ± 0.19 ^a^	1.09 ± 0.07 ^b^	3.61 ± 0.13 ^a^	1.24 ± 0.01 ^b^	3.77 ± 0.21 ^a^
*Total flavan-3-ols*	10.99 ± 0.79 ^c^	17.67 ± 0.48 ^a^	10.66 ± 0.23 ^c^	15.60 ± 0.71 ^b^	11.16 ± 0.20 ^c^	16.73 ± 0.36 ^ab^
***Total phenol***	27.18 ± 2.33 ^c^	39.03 ± 0.97 ^a^	28.11 ± 0.59 ^c^	36.23 ± 2.19 ^b^	27.15 ± 0.99 ^c^	40.55 ± 0.88 ^a^

Note: Different superscript lowercase letters in the same line represent statistically significant differences (*p* < 0.05).

**Table 4:** The contents of carotenoids in unfermented and fermented peach samples from different raw material methods (μg/100 mL).

Categories	CWP		COP		SWP	
	UPs	FPs	UPs	FPs	UPs	FPs
Zeaxanthin	369.62 ± 39.36 ^a^	238.86 ± 23.53 ^c^	288.28 ± 18.36 ^bc^	338.84 ± 19.88 ^ab^	ND	ND
β-cryptoxanthin	132.75 ± 2.09 ^a^	89.48 ± 8.91 ^c^	93.32 ± 3.30 ^bc^	101.61 ± 6.92 ^b^	ND	ND
β-carotene	1524.78 ± 91.38 ^a^	1115.87 ± 67.82 ^b^	1082.93 ± 14.99 ^b^	1134.89 ± 41.80 ^b^	86.33 ± 3.26 ^d^	79.59 ± 10.20 ^d^
Total carotenoids	2008.44 ± 57.68 ^a^	1444.21 ± 92.53 ^c^	1464.53 ± 0.06 ^bc^	1575.33 ± 68.60 ^b^	86.33 ± 3.26 ^d^	79.59 ± 10.20 ^d^

Note: Different superscript lowercase letters in the same line represent statistically significant differences (*p* < 0.05). ND means none detected in samples.

**Table 5:** Calculation of OAVs of aroma-active compounds in unfermented and fermented peach samples from different raw material methods.

	CAS Number	Odor Threshold (μg/L)	CWP		COP		SWP		Odorant Description
			UPs	FPs	UPs	FPs	UPs	FPs	
***Alcohol***									
3-Mehtyl-1-butanol	123-51-3	1.7 [59]	1.19	2.74	<1	<1	<1	<1	Fruity, banana [60]
Furfuryl alcohol	98-00-0	32 [61]	2.22	<1	2.21	<1	<1	<1	bread-like [61]
Linalool	78-70-6	0.17 [62]	8.32	<1	<1	3.88	<1	<1	Citrus-like, bergamot-like [62]
Benzyl alcohol	100-51-6	120 [63]	<1	2.81	<1	2.70	<1	<1	Floral, rose [63]
***Aldehydes***									
Benzaldehyde	100-52-7	320 [63]	<1	3.87	<1	2.63	<1	<1	Almond [64]
Nonanal	124-19-6	1.1 [53]	4.82	<1	5.43	2.38	<1	<1	Fat, citrus, green [53]
Decanal	112-31-2	5 [65]	2.59	<1	2.47	4.71	<1	<1	Sweet, orange peel, citrus, floral [65]
***Ketones***									
2,3-Butanedione	431-03-8	0.059 [61]	<1	114.24	<1	67.63	<1	45.65	Buttery [61]
***Phenols***									
Methyleugenol	93-15-2	0.068 [66]	22.50	69.26	37.65	57.99	<1	<1	Sweet, clove aroma [67]

## Data Availability

The original contributions presented in the study are included in the article/Supplementary Materials. Further inquiries can be directed to the corresponding author.

## Supplementary Materials

The following supporting information can be downloaded at https://www.mdpi.com/article/10.3390/foods14244303/s1, Table S1: Changes in the content of volatile substances in unfermented and fermented peach samples from different raw material methods (μg/L); Table S2: Component of various VOCs in unfermented and fermented peach samples from different raw material methods (μg/L); Table S3: The retention times and calibration curves of sugars and organic acids; Table S4: The retention time and calibration curves of polyphenols; Table S5: The retention time and calibration curves of carotenoids.

## Abbreviations

The following abbreviations are used in this manuscript:

CWP	Peach fruit crushing into puree with peel
COP	Peach fruit crushing into puree without peel
SWP	Peach fruit squeezing into juice with peel
FPs	Fermented peach sample
UPs	Unfermented peach sample
